# Impact of rare JAK/STAT germline mutations on vaccination-induced innate immune responses in a Tyrolian population

**DOI:** 10.7150/ijbs.124098

**Published:** 2026-01-01

**Authors:** Lothar Hennighausen, Gyuhyeok Cho, Sung-Gwon Lee, Yasmin Caf, Jungwook Kim, Ludwig Knabl, Priscilla A. Furth, Hye Kyung Lee

**Affiliations:** 1 Section of Genetics and Physiology, National Institute of Diabetes and Digestive and Kidney Diseases, US National Institutes of Health, Bethesda, Maryland 20892, USA.; 2 Department of Chemistry, Gwangju Institute of Science and Technology (GIST), Gwangju 61005, Republic of Korea.; 3 Y2L2Science GmbH, Zams, Austria.

## Abstract

Vaccination triggers the release of pro-inflammatory cytokines, the stimulation of the Janus Kinase (JAK) - Signal Transducer and Activator of Transcription (STAT) pathway and the activation of interferon response genes. While some JAK/STAT variants are associated with hematopoietic malignancies, the impact of the vast majority is unknown. Here we identify JAK/STAT germline variants in a Tyrolian cohort, including octogenarians, and link specific rare variants to enhanced vaccine-induced interferon transcriptomic responses. AlphaFold 3 predicted conformational changes in JAK and STATs variants, impacting their interactions and formation of receptor complexes. We also identified co-occurring variants in TYK2 and other modulators of interferon signaling that possibly modify the impact of JAK and STAT variants in the innate immune response. Our results demonstrate that the vaccine-induced innate immune transcriptomic response can be used for an *in vivo* functional assessment of mutations controlling key genetic pathways in the innate immune response.

## Introduction

Single nucleotide polymorphisms (SNPs) are estimated to be as frequent as one in every one thousand nucleotides but only a subset of them are non-synonymous SNPs that alter a protein's amino acid sequence. While certain variants have been thoroughly researched and associated with distinct phenotypic traits, the majority are still categorized as variants of unknown significance (VUS). It appears that these variants frequently do not act in isolation but rather function within complex genetic pathways, making it essential to evaluate their effects within their native genetic environment 1. Their influence may not always be evident under basal conditions but, instead only become evident with stress, such as hormonal fluctuations during pregnancy or immune system activation following vaccination or infection. Here we focused on a specific signaling pathway, the Janus Kinase (JAK) and Signal Transducers and Activators of Transcription (STATs) pathway, using a specific and uniform stressor across all subjects, namely COVID-19 mRNA vaccination, to explore the impact of non-synonymous SNPs in JAK-STAT pathway genes on immune activation.

The JAK-STAT pathway is key to the immune response to pathogen exposure or hormonal signals. It plays an indispensable role in translating cytokine signals into genetic responses that regulate a range of biological processes, including body growth 2, lactation 3 and hematopoiesis 3-5. Human mutations and experimental mouse genetic studies have elucidated essential functions of these tyrosine kinases and transcription factors 2, 3, 5, 6. Missense mutations occur within all four JAK family members (JAK1, JAK2, JAK3, and TYK2) and in the seven STAT proteins (STAT1, STAT2, STAT3, STAT4, STAT5A, STAT5B and STAT6). While some variants, such as JAK2^V617F^, are well-characterized 7-9 and associated with clinical disease, the biological significance of most of missense mutations remains largely unclear. Notably, the biological impact of both germline and somatic mutations could be restricted to specific cell types or defined physiological circumstances, such as pregnancy-induced lactation or cytokine triggered immune responses. Moreover, it is possible that epistatic interactions between proteins within the pathway may modify the impact of different missense mutations.

Assessing the impact of combinatorial SNPs in the JAK/STAT pathway and possible role of epistasis on physiological responses in humans requires experimental approaches that permit the spatial and temporal evaluation of molecular responses induced by pathogens, systemic or local signals. JAKs and STATs are activated within seconds after cytokine exposure leading to stereotyped genetic responses. For example, the rapid activation of interferon-stimulated genes (ISGs), controls processes crucial for T cell activation 10. The cycle of gene activation and return to baseline can be measured over hours to days. Vaccination represents a controlled situation where the time of inoculation provides a reproducible means of evaluating the time course, before and after, immune stimulation. Activation of immune or hormonal response genes is the most direct and sensitive readout of the JAK/STAT transcriptional response 11 and can be used to identify candidate JAK/STAT variants influencing downstream genetic programs 12-15.

In this study we employed a set of RNA-seq data from buffy coat samples that initially were used to monitor the innate immune responses following mRNA vaccination 11 in combination with Whole Genome Sequence (WGS) data also obtained from buffy coat samples. The availability of paired RNA-seq and Whole Genome Sequence (WGS) data from the same subjects provided a unique opportunity to assess the impact of JAK/STAT mutations on vaccine-induced activation of interferon-stimulated genes (ISGs) within their natural genetic environment. Limited size studies such as this enable definitive identification of variants within the genome across all JAKs and STATs so that possible epistatic interactions between variants can be biologically explored. We used RNA-seq and WGS data to identify individuals carrying missense mutations in the four *JAK* genes and seven *STAT* genes and investigated their potential impact on vaccine-induced transcriptomic responses. Using AlphaFold3, we also predicted the impact of these mutations on protein structures and predicted function. Findings presented expand our understanding of the structural and molecular impact of specific JAK/STAT variants in vaccine-induced innate immune responses.

## Results

**Figure 1:**
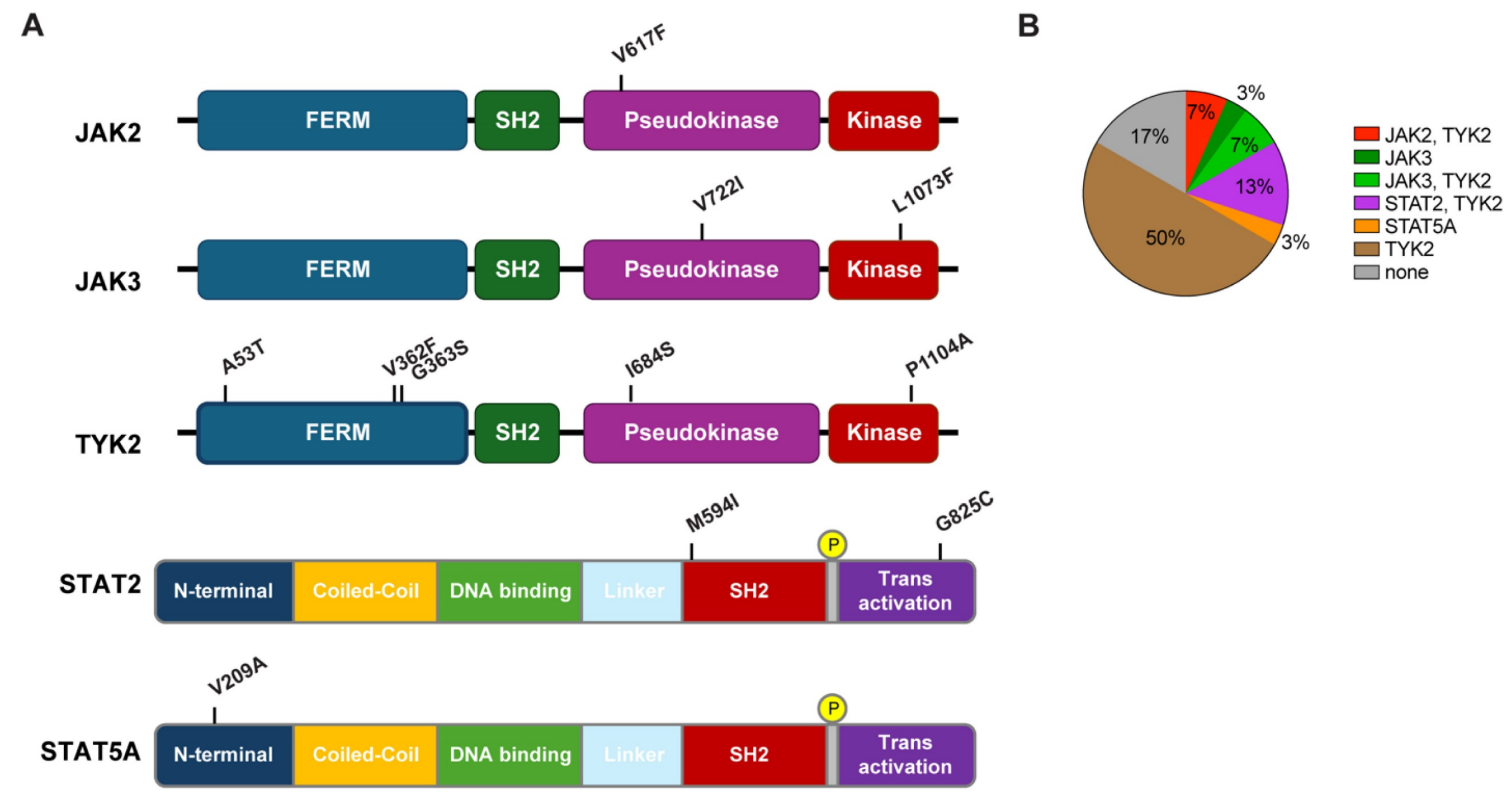
**Mutations in the JAK and STAT genes identified in the Tyrolian cohort. (A)** Schematic representation of human JAK and STAT protein domains, highlighting the locations of mutations found in 30 study participants from the Tyrolian cohort. **(B).** Distribution of individual and combinatorial mutations within the cohort.

Molecular innate immune responses in a Tyrolian cohort following immunization vary 11. Here we hypothesized that variant patterns of transcription following mutation may be related to mutations in JAK/STAT signaling components. To interrogate this possibility, we searched for single nucleotide polymorphisms (SNPs) in protein coding sequences using RNA-seq data from all 30 study subjects of the Tyrolian cohort. Missense mutations were identified in the *JAK2*, *JAK3* and *TYK2* genes as well as in the *STAT2* and S*TAT5A* genes (Figure 1A; Supplementary Table 1). WGS was used to validate these mutations. The allele frequencies (AF) of these germline mutations, based on the GenomAd and AllofUs databases, ranged from approximately 4x10^-4^ for the rare JAK3^L1073F^ and JAK2^V617F^ variants to 2x10^-1^ for the common TYK2^V362F^ mutation (Supplementary Table 1). Mutations in JAKs and TYK2 were identified in the FERM domain (TYK2^A53T^, TYK2^V362F^ and TYK2^G363S^), the pseudokinase domain (JAK2^V617F^, JAK3^V722I^ and TYK2^I684S^) and the kinase domain (JAK3^L1073F^). STAT mutations were positioned in the STAT2 SH2 domain (STAT2^M594I^), the transactivation domain (STAT2^G825C^) and the N-terminal region of STAT5A (STAT5^V209A^).

To assess the potential pathogenicity of the JAK/STAT variants, we employed the COSMIC and the NIH ClinVar database as well as several *in silico* prediction tools (Supplementary Table 2). Except for TYK2^A53T^, all these germline mutations were found in the COSMIC database, suggesting they are also present as somatic activating mutations. The most prominent variants in COSMIC were JAK2^V617F^ with more than 49,000 entries and JAK3^V722I^ with 67 cases. ClinVar categorized only JAK2^V617F^ as likely pathogenic and the JAK3^L1073F^ variant as uncertain significance. All other mutations are considered benign or likely benign. AlphaMissense 16, a state-of-the-art computational tool, predicted pathogenic scores (0.56-1) for TYK2^I684S^ and TYK2^P1104A^, and all other variants, including the JAK2^V617F^ variant, scored in the benign range (0.04-0.34). In contrast, PolyPhen2 17 analysis predicted five variants as deleterious, including TYK2^P1104A^, which also had the highest pathogenic score in AlphaMissense. The REVEL (Rare Exome Variant Ensemble Learner) 18 were in general neutral or benign. Not surprisingly, different tools provided a range of pathophysiological predictions for the mutants, creating a challenge to identify their *in vivo* function in guiding innate immune responses following vaccination.

**Figure 2:**
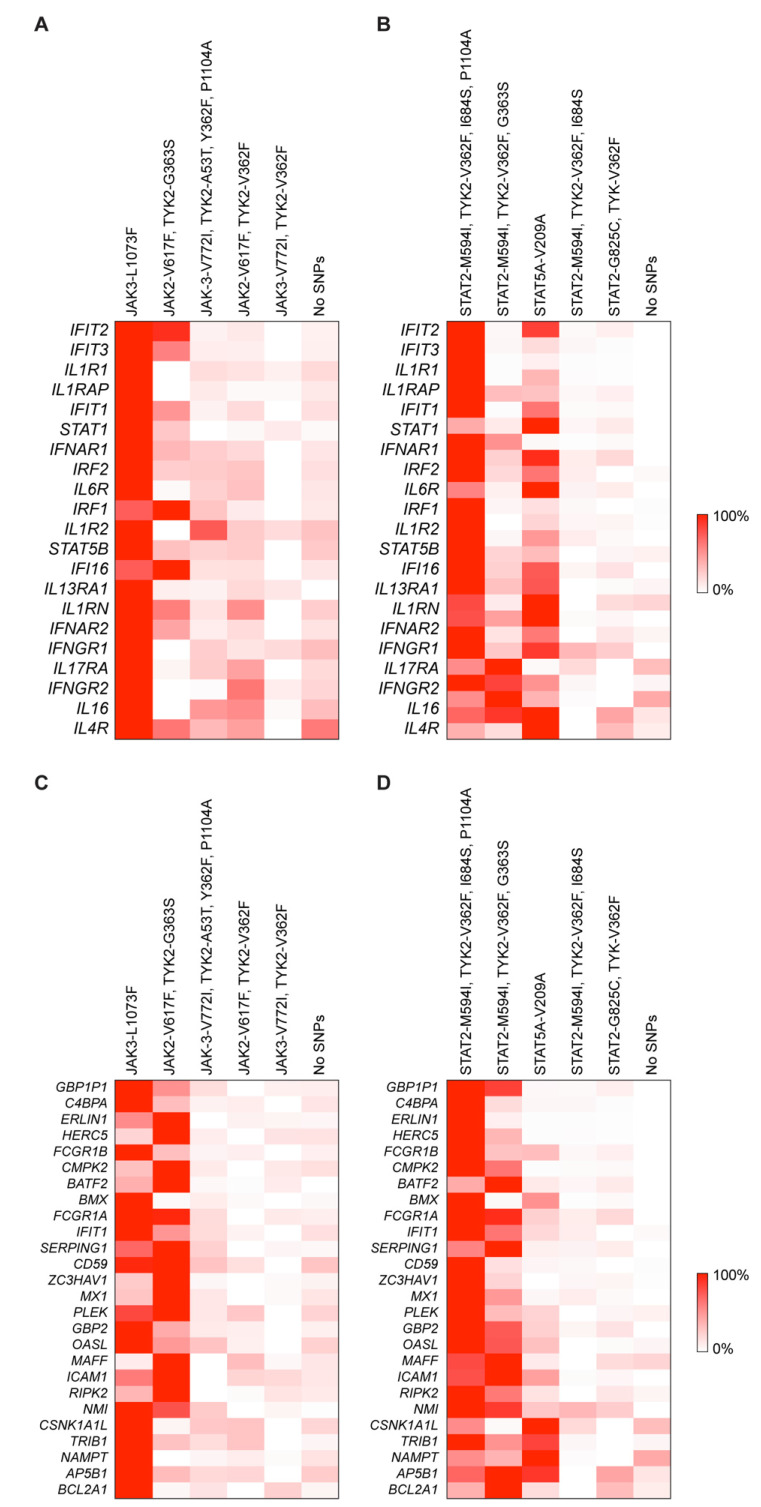
** Transcriptional response of JAK/STAT target genes in patients with mutations following COVID-19 vaccination.** The heatmap shows the relative activity in upregulated interferon-stimulated genes between pre-vaccination and Day 1 post-vaccination for JAK mutations (A and C) and STAT mutations (B and D).

Out of the 30 individuals, five did not carry any missense mutations in any of the JAK/STAT components (Figure 1B). Two individuals carried the polycythemia vera mutation (PV) JAK2^V617F^ mutation, two carried the JAK3^V722I^ mutation and one carried the rare JAK3^L1073F^ variant. To assess if individuals carrying any of these JAK/STAT variants exhibit an altered innate immune response following vaccination with BNT2b2, we analyzed their transcriptomic data obtained from buffy coat prior to vaccination (day 0) and one day following vaccination (day 1) 11. A set of 20 bona fide JAK/STAT targets, including interferon-induced genes that encode antiviral proteins, interferon receptors and transcription factors was used as reference. The strongest response was observed in the individual with the very rare JAK3^L1073F^ variant 19-21 (Figure 2A, Supplementary Table 3). The specific functional consequences of this mutation have not been studied yet 19-21, but its location within the kinase domain suggests it could significantly impact JAK3's activity. The second strongest activation was observed in subject 9, an individual with idiopathic myelofibrosis who carries the JAK2^V617F^ gain of function (GOF) variant 8, 22 and the TYK2^V362F^ variant (Figure 2A, Table 1, Supplementary Table 3). Subject 10 also carried the PV JAK2^V617F^ variant but together with the TYK2^G363S^ variant. Subject 10 demonstrated a mitigated response, suggesting a differential impact of the two TYK2 mutations on the JAK2^V617F^ variant. Two individuals carried the JAK3^V722I^ variant, which is known to be a GOF variant 23, 24, with one individual also carrying TYK2^A53T^, TYK2^V362F^ and TYK2^P1104A^ variants (Table 1, subject 2). A second individual (Table 1, subject 26) carried the TYK2^V362F^ variant. Only subject 2 exhibited an enhanced transcriptomic response suggesting that the JAK3^V722I^ variant by itself is not a strong activating mutation in setting studied here. Three individuals carried the STAT2^M594I^ mutation. It appeared that when the co-mutations TYK2^V362F^ and TYK2^I684S^ were present, there was reduced transcriptional induction. In contrast when co-mutations TYK2^G363S^ or TYK2^P1104A^ were present, there was an enhanced transcriptional immune response. It is possible that specific TYK2 mutations may act independent drivers or co-inducers of STAT2^M594I^, (Figure 2B, Supplementary Table 3). Notably, the response of the one individual also carrying the TYK2^V362F^, TYK2^I684S^ and TYK2^P1104A^ variants (Table 1) exceeded that of the other two individuals carrying different TYK2 variant combinations. Based on these results, we suggest that the TYK2^P1104A^ mutation, located in the kinase domain, enhances the transcriptomic response triggered by the STAT2^M594I^ variant, which is within the SH2 domain. Additional missense mutations were also identified in the *STAT2* and *STAT5A* genes. In addition to the three individuals carrying the STAT2^M594I^ variant, discussed above, individuals carrying either the STAT2^G825C^ variant or a STAT5A^V209^ variant were identified (Figure 1, Table 1). None of these variants have been reported to be activating ones. An enhanced transcriptomic response was observed in the individual carrying the STAT5A^V209A^ mutation but not in the STAT2^G825C^ proband. Transcriptomic responses are presented in Figure 2C-D and Supplementary Table 3.

### Co-existing mutations impacting interferon signaling

Besides modulating mutations in the core JAK/STAT components shown here, mutations in upstream receptors and other regulatory components are likely to impact cytokine signaling and thus vaccine-induced innate immune responses. To further explore this possibility, we identified additional missense mutations in probands carrying JAK/STAT mutations (Table 1). Missense mutations were identified in the IFN-α receptors 1 and 2 (IFNAR1 and IFNAR2), the IFNGR2, the IFNLR1, receptors critical for the response to viral infections (Table 1). We also identified the IL6R^D358A^ variant in individuals carrying the JAK3 and STAT2 mutations. This variant is associated with decreased treatment response to tocilizumab and worse outcomes in giant cell arteritis by enhancing CD4 T cell response to IL-6 25.

Additional mutations were found in DHX58 (LGP2 26), a protein required for controlled innate immune responses. Mutations were also identified in the gene family encoding oligoadenylate synthetases (OAS), which also modulate the innate immune response.

### Structural consequences of variants

**Figure 3:**
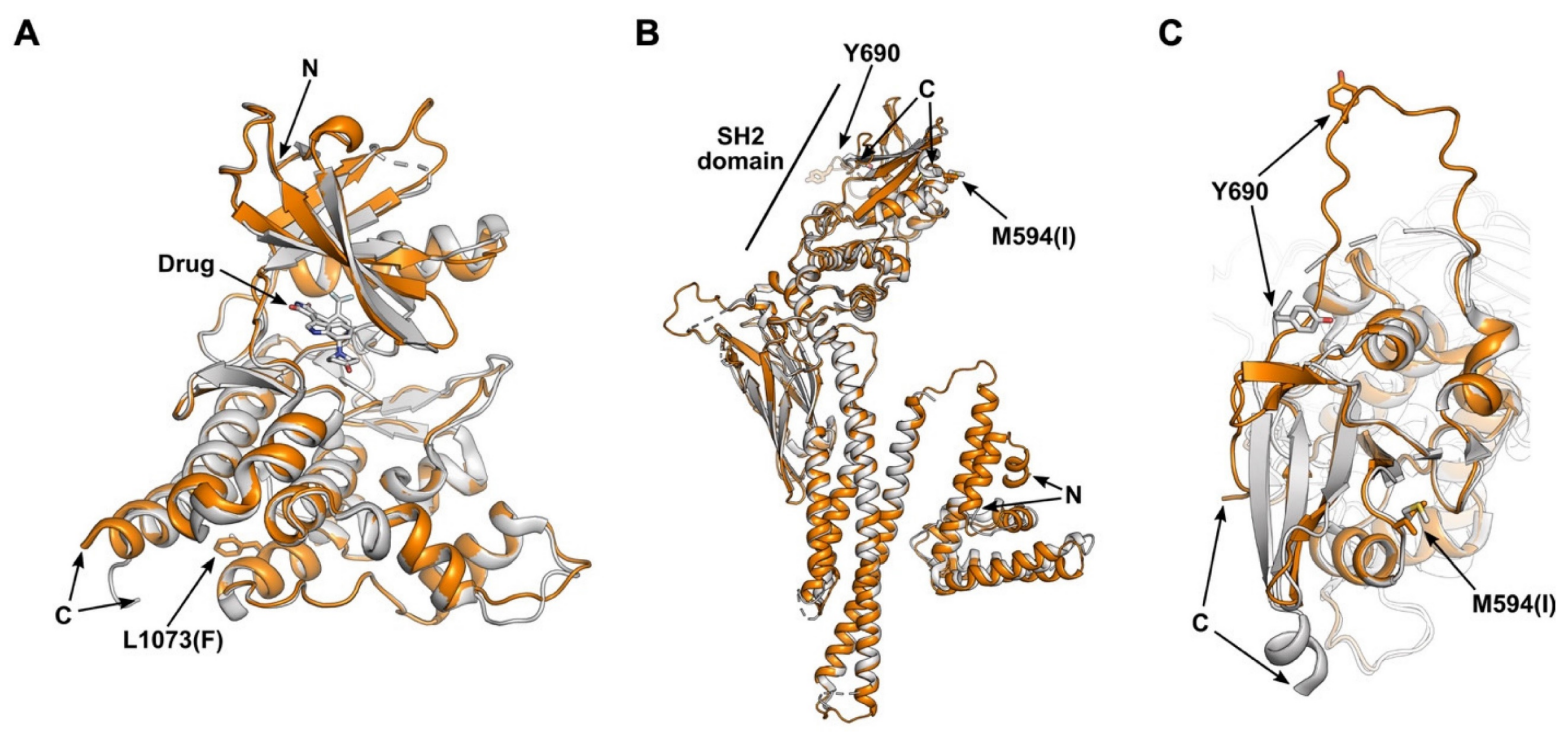
**Predicted structural impact of mutations on the JAK-STAT pathway. (A)** Superimposition of the JAK3 kinase domain (residues 812-1124) from the wild-type crystal structure (gray; PDB: 3PJC) and the AlphaFold 3-predicted L1073F mutant (orange). The bound drug in the ATP-binding site and the side chains of residue L1073(F) are depicted as sticks. **(B)** Superimposition of STAT2 wild-type (gray; PDB: 6UX2) and the AlphaFold-predicted M594I mutant (orange). The wild-type structure (residues 1-713) is derived from a crystal structure of STAT2 complexed with the Zika virus NS5 protein on the coiled-coil domain. **c.** Zoomed-in view of the SH2 domain from panel (b).

Next, we used AlphaFold3 27, 28 to predict the structural impacts of the JAK and STAT mutations in this study cohort. Given the significant immune response associated with a single amino acid substitution, the JAK3^L1073F^ mutation was selected as a primary target for investigation. This mutation, located in the kinase domain of JAK3 4, was examined using AlphaFold 3 28. The only experimentally validated structure of wild-type JAK3 is the crystal structure of its kinase domain (residues 812-1124) complexed with an inhibitor at the ATP binding site (PDB: 3PJC) 29. To assess the impact of the JAK3^L1073F^ mutation, we predicted the structure of the mutant JAK3 kinase domain (residues 812-1124) (Figure 3A). Superimposition of the predicted mutant structure with the wild-type crystal structure yielded a very low root mean square deviation (RMSD) of 0.415 Å. Notably, the L1073F residue is located approximately 25 Å from the ATP binding site, and no meaningful structural alterations were observed in its local environment, consistent with the conservative nature of a single amino acid change. As the kinase domain is known for phosphorylating substrates, such as STAT proteins, the L1073F mutation might affect the enzymatic kinetics.

Enhanced immune responses were observed in association with the STAT2^M594I^ mutation. The only experimentally validated structure of wild-type STAT2 is the crystal structure of STAT2 (residues 1-713) in complex with the non-structural protein 5 (NS5) of Zika virus (PDB: 6UX2) 30. Because NS5 binds to the coiled-coil domain and the M594I mutation is located in the SH2 domain 4, our analysis focused on comparing the structural changes in the SH2 region. We predicted the STAT2^M594I^ mutant spanning residues 1-713 (Figure 3B-C). The overall structures of wild-type and mutant STAT2 are highly similar, with an RMSD of 0.488 Å. However, local conformational differences near the M594(I) residue are observed. When STAT2 binds to receptor-associated JAK family proteins (JAK1 and TYK2), phosphorylation of tyrosine 690 (Y690) within the SH2 domain occurs 31. In the WT structure, Y690 is positioned 26 Å away from the M594 residue, and the loop containing Y690 is rigid and forms a β-sheet with the SH2 domain. In contrast, in the M594I mutant, Y690 shifts to a position 21 Å away, and the loop containing Y690 loses its secondary structure. Given that Y690 phosphorylation is essential for the heterodimerization of STAT2 with STAT1 32, 33, these conformational changes would be predicted to affect heterodimer formation and downstream signal transduction.

### Germline versus somatic mutations

The question arose whether the mutations were of germline or somatic origin. Since we did not have access to the genomes of parents or children of individuals carrying mutations (one half of the study participants were elderly nuns), it was not possible to investigate the heritable nature of the mutations, the definitive proof that the mutations were carried in the germline. We therefore drew on molecular features of the RNA-seq and WGS datasets obtained from buffy coat and analyzed the reads covering each mutation (Supplementary Figure 1; Supplementary Table 4). Buffy coat consists mainly of white blood cells and mutations detected are generally considered of germline, but they can also include somatic mutations, namely those from clonal hematopoiesis 34. First, we investigated the sequencing reads obtained from the WGS analyses. If a mutation were germ-line one would postulate that in the hemizygous state approximately 50% of the reads would represent the mutant and approximately 50% would represent wild-type. The number of reads covering the individual mutations ranged from 24 to 53 (Supplementary Table 4). The ratio of reads carrying the wt and mutant nucleotides ranged from 40% to 60% (Supplementary Figure 1; Supplementary Table 4). This finding is consistent with a germline origin of the mutations. We extended this analysis to the RNA-seq data as we had at last three independent samples from each individual. The read counts for each mutant site, except for JAK2^V617F^, for each individual ranged between 200 and more than 3600 and the ratio of wild type and mutant reads was approximately 50%, supporting the notion that the mutations are germline. Somatic mutations leading to clonal hematopoiesis would have yielded a skewed ratio. This scenario is visible in the two individuals carrying the JAK2^V617F^ variant. While the ratio of wild type to mutant alleles in individual #10 was approximately 75% to 25%, it was the reverse in individual (#9 Supplementary Table 4). WGS data was available for only one of the individuals due to the other individual being deceased. The WGS data available showed a 40 (WT) -60 (mutant) ratio. This is compatible with a germ-line origin although not proof. The more dramatically skewed ratios in the RNA expression could be due to either alterations in specific cell subset numbers (e.g. clonal expansion) or RNA expression levels changes due to the mutational profile. Examination of specific cell subsets associated with mutational screening could be a next step to investigate the source of the mutation and reasons for the skewed RNA expression profiles.

## Discussion

Vaccination triggers an immune response, leading to the release of pro-inflammatory cytokines, the induction of global transcriptional changes in whole blood and the activation of interferon (IFN)-inducible genes (ISGs) within 12-24 hours 11, 35, 36. Here we demonstrate that individuals carrying the very rare JAK3^L1073F^, the rare STAT2^M594I^ and STAT5A^V209A^ variants exhibit an enhanced innate immune response following BNT2b2 mRNA vaccination as evidenced by the excessive activation of ISGs. Although we could not detect a clear effect of STAT2^M594I^ alone, we observed strong synergistic effects when it co-occurred with TYK2^P1104A^ or TYK2^G363S^, particularly in combination with JAK2^V617F^ or STAT2^M594I^ variants, but not with TYK2^V362F^ or TYK2^I684S^. Unlike STAT2^M594I^, TYK2^I684S^, and TYK2^P1104A^, we observed no increase in transcriptional activity in individuals carrying JAK3^V722I^, TYK2^A53T^, and TYK2^V362F^. This suggests that JAK3^V722I^ and/or TYK2^A53T^ may act as potential inhibitors. In contrast, TYK2^I684S^ could support the effect of TYK2^P1104A^. As of now, there are no documented reports in the scientific literature on these variants and their GOF capacity. Individuals carrying JAK/STAT mutations also carry TYK2 variants and mutations in upstream receptors and other components of interferon signaling, opening the possibility of epigenetic synergy between a range of regulatory proteins. While mutations in the JAK/STAT pathway are known to independently contribute to disease pathogenesis 6, 9, 37-39, specific studies focusing on their combined or synergistic effects are scarce. Here we show that an aberrant immune response can be modified by the combined consequence of mutations in more than one regulatory component of the JAK/STAT pathway, including interferon and interleukin receptors.

Each of the mutations studied here have disease associations reported in the literature (Supplementary Table 2). The JAK2^V617F^ mutation is associated with PV. Significantly, the two individuals carrying the JAK2^V617F^ variant support the concept that the impact of JAK/STAT mutations can be modulated by mutations in other genes that are part of the regulatory program. The stronger response was seen in the individual with PV carrying the additional TYK2^G363S^ variant (Table 1, Subject 10) with a weaker response in the individual with idiopathic myelofibrosis who carried an additional TYK2^V362F^ variant (Table 1, Subject 9). Both the TYK2^G363S^ and TYK2^V362F^ variants are independently associated with development of myeloproliferative neoplasms 40, 41. Two subjects with the Stat2^M594I^ variant carry hematologic diagnoses, myelodysplastic syndrome (Table 1, Subject 4) and monoclonal gammopathy of undetermined significance (MGUS) (Table 1, Subject 16).

IFNAR1^V168L^ and the IL6R^D358A^ have been linked to inflammatory and autoimmune diseases 25 but none of the subjects carrying these variants reported any autoimmune or inflammatory disease. Two of the four subjects carrying the IFNAR1^V168L^ variant demonstrated hematologic disease (Table 1, Subjects 10 and 4) and two of the four subjects carrying the IL6R^D358A^ variant also demonstrated hematologic disease (Table 1, Subjects 4 and 16). It is noted that Subject 4 demonstrated the highest activation found in this study.

JAKs and TYK2 form both homodimers and heterodimers when cytokine receptor signaling is activated, with their proximity permitting trans-phosphorylation and activation. TYK2 is known to pair with both JAK1 and JAK2 in several cytokine signaling pathways 42-45, and the formation of heterodimers is thought to be fundamental for normal immune function, with therapeutic implications. While the published literature focuses on the impact of individual mutations it is necessary to consider the combined consequences of mutations in JAKs and TYKs. This is exemplified in four individuals that carry both JAK and TYK2 variants in different combinations.

Among the two STAT2 and one STAT5A variant, only the STAT2^M594I^ variant was associated with an enhanced transcriptomic response. At present, this mutation has not been definitively associated with disease. However, the three individuals in our study cohort carrying this variant exhibited distinctly different transcriptomic responses suggesting a modulatory impact of the different combinations of TYK2 mutations. Specifically, while the more common TYK2^V362F^ mutation by itself does not appear to enhance the innate immune response upon vaccination, it might synergize with the JAK2^V617F^ and STAT2^M594I^ variants. STAT2 is a pivotal component of the IFN-I signaling pathway and upon activation, it initiates the transcription of interferon-stimulated genes (ISGs). STAT2 GOF mutations, such as STAT2^R148Q^ and STAT2^A219V^ cause unrestrained IFN-I signaling and have been linked to type I interferonopathy 46, 47.

From a structural perspective, the STAT2^M594I^ variant is predicted to increase the flexibility of the loop containing Y690, a key phosphorylation site, potentially enhancing its accessibility for JAK-mediated phosphorylation and downstream signal transduction. In contrast, the isolated JAK3 kinase domain bearing the L1073F mutation shows only minimal structural changes. However, given the strong immune response observed in the patient carrying this mutation, its impact may be more pronounced within the signaling complex. Indeed, our *in silico* models of the JAK3 complex with IL-2 family receptors 48 (Supplementary Figure 2) showed that while wild-type JAK3 forms an auto-phosphorylated conformation with JAK1, the L1073F mutation shifts the equilibrium toward an auto-inhibitory state. A similar trend was observed in the IL-7 complex predictions. The presence of distinct auto-phosphorylated and auto-inhibitory conformations within the JAK family has been well documented 45. These divergent outcomes suggest that while the L1073F substitution does not substantially alter the structure of the isolated kinase domain, it may influence the conformational state and regulatory behavior of JAK3 within multi-protein complexes. Although these predictions require experimental validation, they imply that the mutation could disrupt normal complex formation and regulatory interactions, with significant downstream effects on signaling.

The vaccine-induced innate immune response, characterized by the excessive activation of ISGs, is controlled by the extended JAK/STAT machinery, which includes, but is not limited to, interferon and interleukin receptors, ligands as well as negative regulators and modulators such as DHX48 and OAS members. Very rare mutations and common mutations in these components were found in our study participants, and it can be hypothesized that the compendium of missense mutations defines the innate immune response. Since many of these mutations are of unknown significance their impact on interferon responses cannot be predicted.

Some JAK/STAT mutations have been associated with myeloproliferative neoplasms (MPNs) that are more common in older people, reflecting a cumulative impact over time of otherwise weakly activating mutations. Our study cohort included 16 octogenarians and 14 individuals that were on average 25 years younger, and the most profound transcriptome responses were observed in the octogenarians. Thus, the impact of variants of unknown significance, such as JAK3^L1073F^, might reveal itself preferentially in older individuals. Thus, for studies like ours, there should be an emphasis on recruiting all age groups, particularly the elderly.

Our findings highlight the combinatorial complexity of JAK/STAT mutations in humans that likely impact their physiology and pathology and underscore the importance of genome-wide analyses for understanding the impact of specific single mutations present. Since mutations in humans do not exist in isolation, their impact can only be gauged in the context of additional mutations in their native setting. Understanding the complexity of epistatic SNP interactions has become a major challenge 1 in understanding how the co-presence of several variants can modulate physiological and pathophysiological responses.

A limitation of the investigation is that the individuals studied all received an mRNA vaccine and the question could arise as to whether results would be generalizable to other types of vaccines, including but not limited to live-attenuated, inactivated, subunit, and viral vector. In fact, the specific transcriptional responses to an individual vaccine do vary according to type of vaccine as well as other factors such as exposure history and vaccine schedule. The question of how specific mutational profiles influence responses from different classes of vaccines as well as different antigens would need to be part of a broader study. Here we deliberately focused on individuals receiving with known exposure histories and a single vaccine type and schedule to minimize variability.

## Materials and Methods

### Ethics

The Institutional Review Board (IRB) of the Office of Research Oversight/Regulatory Affairs, Medical University of Innsbruck, Austria (EK Nr: 1064/2021) approved the study. This IRB has the responsibility for the reviewing all human research studies performed in the State of Tyrol (Austria). All subjects provided written informed consent, and all subject information was coded and anonymized prior to analysis.

### Whole genome sequencing and data analysis

Genomic DNA was isolated from human buffy coat samples using the Wizard Genomic DNA Purification Kit (Promega). PCR-free libraries were prepared from 1µg of genomic DNA utilizing the TruSeq DNA PCR-Free HT Sample Preparation Kit (Illumina). The median library fragment size was ~400 base pairs (bp). Unique dual-index DNA barcodes were incorporated to allow pooling while minimizing barcode hopping. Pooled libraries were sequenced on the NovaSeq 6000 or NovaSeqX+ (Illumina) platforms, generating a minimum of 300 million paired-end reads (151 base pairs each) per library. Sequencing was performed at the NIH Intramural Sequencing Center (NISC).

### Protein structure prediction and analysis

The JAK/STAT structure was predicted using AlphaFold3 (https://alphafoldserver.com). Structural analysis and visualization were conducted with PyMOL (version 2.4.1) and ChimeraX (version 1.8).

## Supplementary Material

Supplementary materials and methods, figures and tables.

## Figures and Tables

**Table 1:**
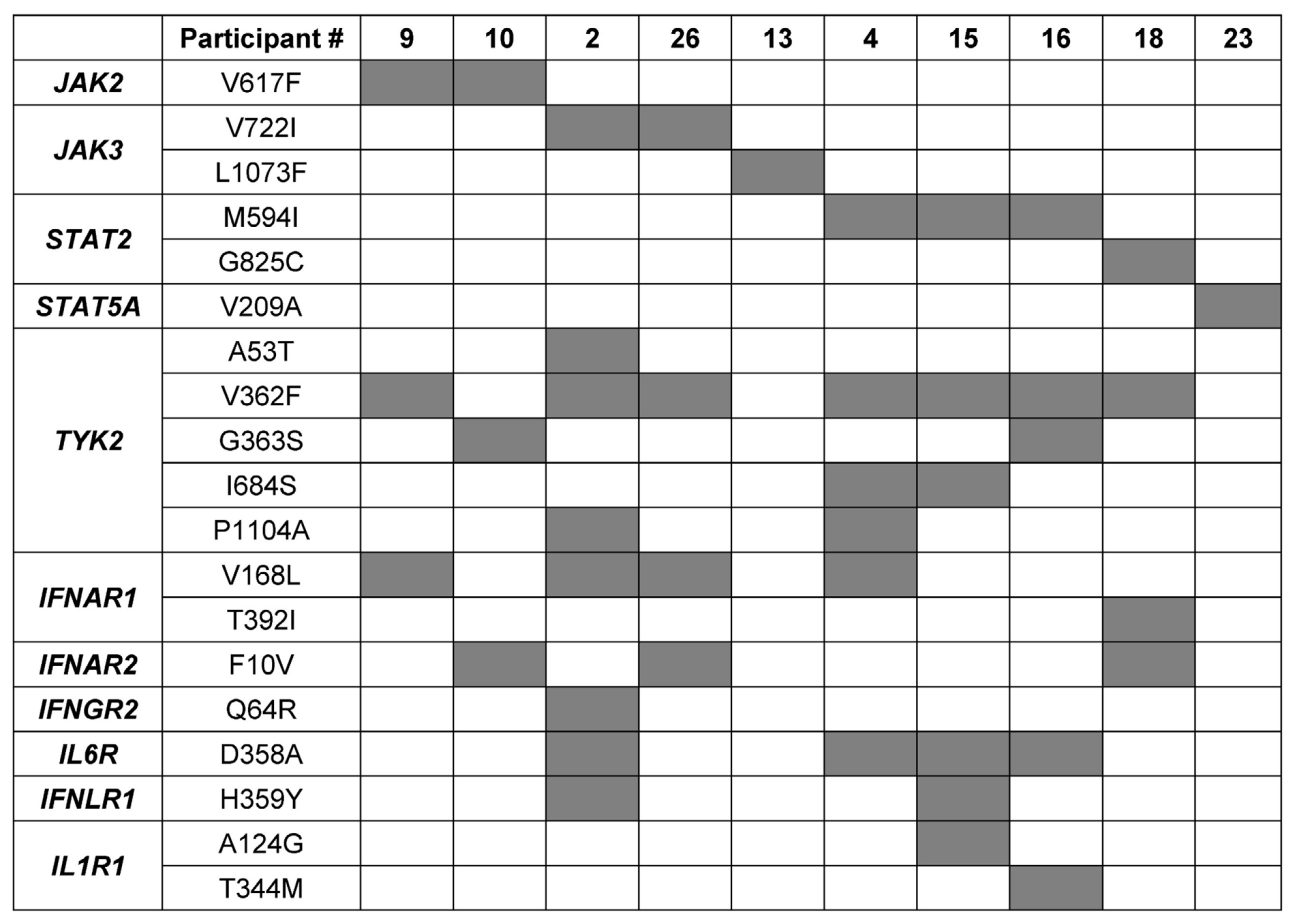
List of JAK and STAT mutations in individuals of the cohort.
