# Supplementary material for: Impact of rare JAK/STAT germline mutations on vaccination-induced innate immune responses in a Tyrolian population
Source: Int J Biol Sci. 2026 Jan 1;22(2):519–28. doi: 10.7150/ijbs.124098 (PMC12780842; doi:10.7150/ijbs.124098)
Supplement: Supplementary file 1 — Supplementary materials and methods, figures and tables. [file ijbsv22p0519s1.pdf]

Supplementary information

Impact of rare JAK/STAT germline mutations on vaccination-induced innate immune responses in a Tyrolian population

Materials and Methods

Whole genome sequencing data analysis

Data analysis was performed using the Parabricks Germline Pipeline (v4.0.1) for germline mutation detection (https://docs.nvidia.com/clara/parabricks/4.0.1/whatsnew.html). Sequencing reads were aligned to the human reference genome (hg38) with BWA-MEM[1]. Duplicate reads were marked with Picard tools[2]. The Genome Analysis Toolkit *(*GATK*)* was used for base quality score recalibration (BQSR) and variant calling. Variants were called with HaplotypeCaller in gVCF mode, applying a minimum Phred-scaled confidence threshold of 16.

SNP detection in RNA-seq data

Raw RNA-seq data were subjected to quality control with *FastQC* (v0.11.9) (https://www.bioinformatics.babraham.ac.uk/projects/fastqc/). Adapter trimming and quality filtering were performed using Trimmomatic[3] (version 0.36). Reads were aligned to the hg19 reference genome with the STAR two-pass procedure[4] (version STAR 2.7.9a). Aligned reads were further processed with BWA MEM[1] (version 0.7.15) and duplicates were marked using Picard tools[2] (version 2.9.2).

Variant calling followed the GATK workflow:

(i) base recalibration with BaseRecalibrator*,* AnalyzeCovariates, and PrintReads using dbSNP138 (provided by NIH Biowulf);

(ii) variant calling with HaplotypeCaller in “discovery” genotyping mode, generating gVCFs with a minimum Phred-scaled confidence threshold of 30.

Variants were filtered using hard thresholds: QD < 2.0, QUAL < 30.0, SOR > 3.0, FS > 60.0, MQ < 40.0, MQRankSum < –12.5, and ReadPosRankSum < –8.0. Additional filtering excluded variants overlapping repetitive elements[5] (UCSC masked and simple repeats; https://hgdownload.soe.ucsc.edu/goldenPath/hg19/database/) and ENCODE blacklisted regions[6] (https://mitra.stanford.edu/kundaje/akundaje/release/blacklists/hg19-human/). Only heterozygous (0/1) or homozygous alternate (1/1) SNVs were retained. Further criteria included: read depth ≥10, exclusion of excessive read depth ($d + 3\sqrt{d}$,

where d = mean depth)[7] (d+3√d, d = average read depth), and allele frequency ≥10%. Filtering was performed using BEDtools (version 2.26.0), BEDOPS (version 2.4.3) and VCFtools (version 0.1.17)[8-10]. SNVs within ±5 bp of indel borders were discarded as likely false positives. SNP positions were subsequently lifted over from hg19 to hg38.

mRNA sequencing (mRNA-seq) data analysis

Raw mRNA-seq data underwent QC with FastQC (v0.11.9). Reads were processed with Trimmomatic[3] (version 0.36) and aligned to hg19 using STAR[4] (version STAR 2.7.9a) in paired-end mode (150 bp). Gene-level counts were obtained using HTSeq[11] (version 0.9.1). Normalization and differential expression analysis were performed with the DESeq2 package[12] in R (https://www.R-project.org/)[13]. Confounding factors were removed using RUVSeq[14]. Pre-filtering retained only genes with ≥10 total reads. Visualization was performed with dplyr (https://CRAN.R-project.org/package=dplyr) and ggplot2[15]. Statistical testing employed a paired, two-sided Wilcoxon test. P-values were adjusted using the Benjamini–Hochberg method, with a false discovery rate threshold of pAdj ≤ 0.05.

Supplementary Figures

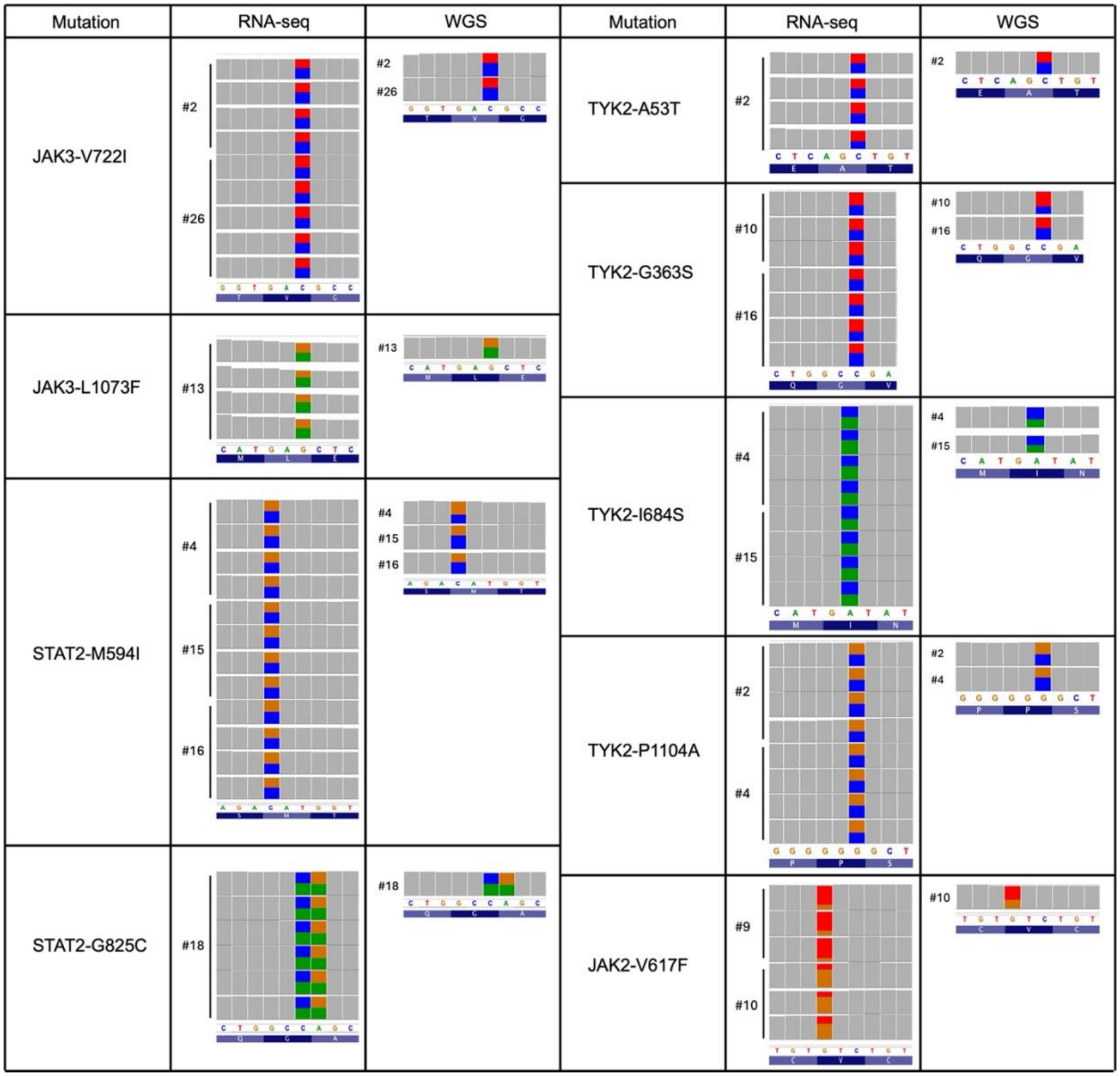

Supplementary figure 1. Sequence alignments from RNA-seq and WGS data of individuals harboring a SNP.

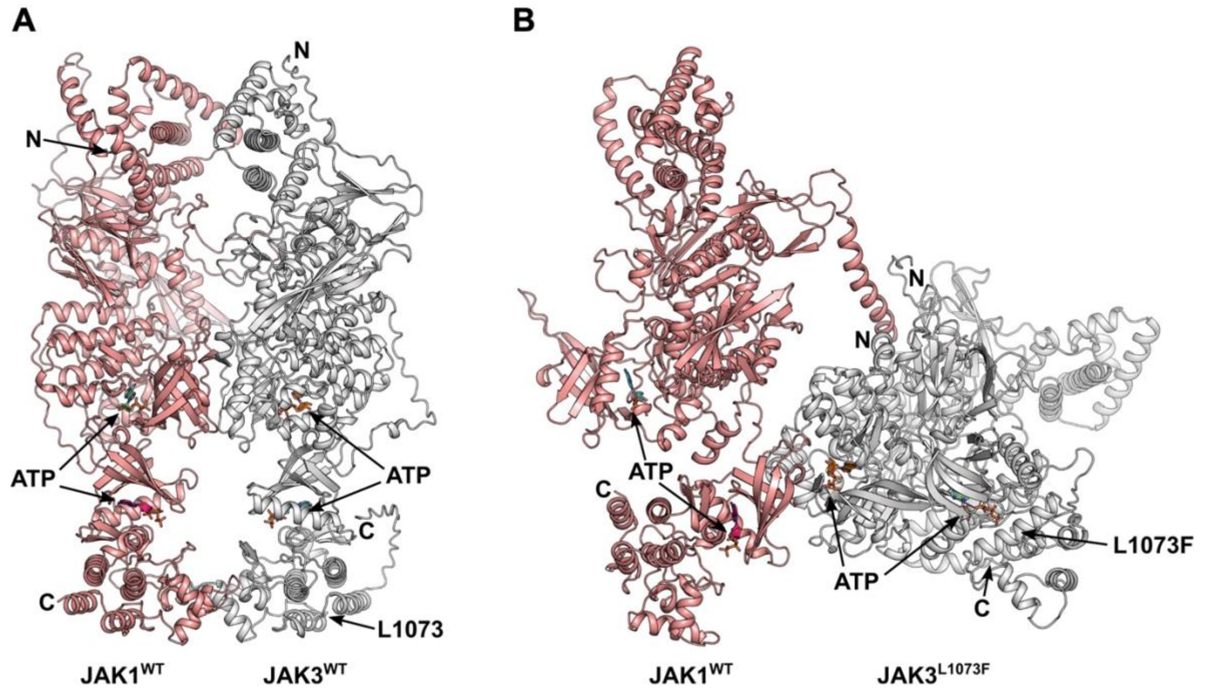

Supplementary figure 2. AlphaFold3-predicted models of the IL-2-mediated signaling complex. Shown as cartoon representations, the complexes containing (A) WT JAK3 and (B) the L1073F mutant are depicted. For clarity, the IL-2 signaling complex components (IL-2 and its receptor) and STAT5A are omitted.

Supplementary Table 1. JAK and STAT mutations identified in 30 study subjects of the Tyrolian cohort

Gene	AA substitution	rsID	gnomAD		All of Us	
			Allele Count	Allele frequency	Allele Count	Allele frequency
JAK2	V617F	rs77375493	471	2.93E-04	278	5.67E-04
JAK3	V722I	rs3213409	15743	9.75E-03	4480	9.13E-03
	L1073F	rs200580168	533	3.31E-04	107	2.18E-04
TYK2	A53T	rs55762744	14980	9.29E-03	3486	7.10E-03
	V362F	rs2304256	446002	2.76E-01	116321	2.37E-01
	G363S	rs2304255	113510	7.03E-02	27192	5.54E-02
	I684S	rs12720356	10	7.97E-02	30076	6.13E-02
	P1104A	rs34536443	59598	3.71E-02	13893	2.83E-02
STAT2	M594I	rs2066807	93983	5.82E-02	23543	4.80E-02
	G825C	rs61754170	29650	1.84E-02	5846	1.19E-02
STAT5A	V209A	rs2230123	6766	4.19E-03	8973	1.83E-02

Supplementary Table 2. Potential clinical pathogenicity of JAK and STAT mutations.

Gene	AA substitution	rsID	COSMIC	ClinVar	*In silico* pathogenicity score			Literature-Reported Disease Associations
			case #	Clinical significance	Alpha Missense	PolyPhen2	REVEL	
JAK2	V617F	rs77375493	>49,000	Likely pathogenic	0.33	0.93	0.88	Myeloproliferative neoplasms[16,17]
JAK3	V722I	rs3213409	67	Benign	0.07	0.00	0.16	Acute Myelocytic Leukemia[18,19]<br>Natural Killer cell lymphoma[20]<br>T cell lymphoma[21]<br>Idiopathic erythrocytosis[22]
	L1073F	rs200580168	3	Uncertain	0.29	0.98	0.48	Melanocytic Neoplasms[23]
TYK2	A53T	rs55762744	0	Benign	0.16	0.92	0.46	Multiple sclerosis[24]<br>Autoimmunity[25,26]
	V362F	rs2304256	31	Benign	0.08	0.02	0.05	Systemic sclerosis[27]<br>Systemic lupus erythematosus[28-30]<br>Autoimmune rheumatic diseases[31]<br>Psoriasis[32]
	G363S	rs2304255	2	Benign	0.08	0.00	0.03	Psoriasis[33]<br>Cancer metastases[34]<br>COVID-19 susceptibility[35-37]
	I684S	rs12720356	5	Benign	0.59	0.97	0.34	Psoriasis[32,38]<br>Autoimmunity[25,39,40]<br>Systemic sclerosis[27]<br>Rheumatoid arthritis[41]<br>Systemic lupus erythematosus[42]
	P1104A	rs34536443	3	Likely benign	0.83	0.97	0.59	Systemic sclerosis[27]<br>Autoimmunity[25,39,40,43,44]<br>Rheumatoid arthritis[41]<br>Tuberculosis[45]
STAT2	M594I	rs2066807	6	Benign	0.08	0.00	0.36	Cervical Cancer[46]<br>COVID-19 susceptibility[35]<br>Psoriasis[47]
	G825C	rs61754170	1	Benign	0.09	0.01	0.01	Lymphoma[48]<br>Autoinflammatory diseases[49]
STAT5A	V209A	rs2230123	2	Benign	0.08	0.11	0.00	Sickle Cell Anemia with Stroke[50]

Supplementary Table 3. List of genes regulated through the JAK/STAT pathway at Day0 and Day1 after the vaccination in the cohort.

	JAK mutations						
	Fold Change	JAK3 L1073F	JAK2 V617F	JAK3 V722I	JAK2 V617F	JAK3 V722I	no SNP
				TYK2 A53T			
				TYK2 V362F	TYK2 V362F	TYK2 V362F	
			TYK2 G363S				
				TYK2 P1104A			
JAK/STAT targets	IFIT2	11.87	2.65	2.21	11.16	1.56	2.23
	IFIT3	11.13	2.49	2.62	7.27	1.77	2.43
	IL1R1	6.69	1.90	2.03	1.11	1.50	2.12
	IL1RAP	6.26	1.60	1.97	1.49	1.60	2.00
	IFIT1	5.57	2.46	2.04	3.66	1.81	2.38
	STAT1	4.74	1.49	1.39	2.29	1.71	1.46
	IFNAR1	3.50	1.54	1.67	1.92	1.10	1.40
	IRF2	3.47	1.76	1.69	1.66	1.09	1.45
	IL6R	3.39	1.74	1.57	1.10	1.04	1.32
	IRF1	3.29	1.41	1.95	4.06	1.13	1.45
	IL1R2	3.08	1.69	2.62	1.21	1.53	1.75
	STAT5B	2.98	1.52	1.46	1.63	1.04	1.54
	IFI16	2.97	1.54	1.53	3.59	1.18	1.47
	IL13RA1	2.70	1.67	1.51	1.53	1.58	1.43
	IL1RN	2.69	1.88	1.20	2.00	0.97	1.38
	IFNAR2	2.67	1.36	1.23	1.77	1.09	1.31
	IFNGR1	2.63	1.28	1.45	1.08	1.35	1.56
	IL17RA	2.53	1.69	1.39	1.09	1.02	1.29
	IFNGR2	2.16	1.68	0.88	0.86	0.97	1.12
	IL16	1.67	1.32	1.28	0.91	0.93	1.15
	IL4R	1.56	1.23	1.16	1.34	0.96	1.33
enhancer immune genes	GBP1P1	4.85	1.68	1.07	3.02	1.33	1.36
	C4BPA	8.03	1.61	1.75	3.31	1.18	2.03
	ERLIN1	4.11	1.30	1.62	6.55	1.55	1.51
	HERC5	2.19	1.69	1.32	5.48	1.88	1.88
	FCGR1B	10.11	1.72	1.93	3.95	1.26	1.89
	CMPK2	2.51	1.60	1.16	5.66	1.64	1.83
	BATF2	8.10	2.02	1.48	18.97	3.07	1.40
	BMX	9.32	2.34	1.89	1.86	1.68	1.90
	FCGR1A	5.48	1.80	1.06	5.35	1.50	1.41
	IFIT1	5.57	2.46	2.04	3.66	1.81	2.38
	SERPING1	3.03	1.86	1.29	3.79	1.40	1.37
	CD59	2.57	1.48	1.26	2.62	1.02	1.47
	ZC3HAV1	2.05	1.36	1.23	4.47	1.32	1.42
	MX1	1.93	1.60	1.36	3.43	1.41	1.61
	PLEK	3.21	1.56	1.91	3.61	1.30	1.79
	GBP2	4.91	1.74	1.66	2.79	1.38	1.63
	OASL	2.91	1.80	1.47	2.11	1.36	1.71
	MAFF	1.92	1.23	3.62	8.80	1.49	2.12
	ICAM1	2.78	1.32	1.81	3.70	1.77	1.60
	RIPK2	2.12	1.21	1.26	3.79	1.55	1.47
	NMI	2.93	1.69	1.27	2.58	1.35	1.28
	CSNK1A1L	7.46	2.76	2.86	1.36	1.04	2.30
	TRIB1	5.09	2.10	2.53	2.56	1.52	1.68
	NAMPT	9.07	1.78	2.14	1.46	1.60	2.54
	AP5B1	3.28	1.49	1.52	1.79	1.08	1.62
	BCL2A1	3.28	1.65	1.39	1.47	1.82	1.49

	STAT mutations						
	Fold Change	STAT2 M594I	STAT2 M594I	STAT5A V209	STAT2 M594I	STAT2G825C	no SNP
		TYK2 V362F	TYK2 V362F		TYK2 V362F	TYK2 V362F	
			TYK2 G363S				
		TYK2 I684S			TYK2 I684S		
		TYK2 P1104A					
JAK/STAT targets	IFIT2	19.25	5.73	5.10	2.82	2.45	2.23
	IFIT3	12.03	5.17	6.48	2.84	2.49	2.43
	IL1R1	2.70	4.05	1.54	1.99	1.60	2.12
	IL1RAP	4.12	3.43	1.88	1.84	1.35	2.00
	IFIT1	8.41	3.36	6.18	2.75	2.23	2.38
	STAT1	3.19	2.67	5.13	1.69	2.08	1.46
	IFNAR1	3.36	2.26	1.26	1.49	1.26	1.40
	IRF2	2.66	2.13	2.08	1.57	1.50	1.45
	IL6R	1.13	2.09	1.24	1.67	1.28	1.32
	IRF1	3.26	3.00	4.17	1.53	1.92	1.45
	IL1R2	1.97	0.93	1.48	1.17	1.26	1.75
	STAT5B	2.39	1.93	1.45	1.40	1.32	1.54
	IFI16	4.66	1.95	2.35	1.75	1.73	1.47
	IL13RA1	2.11	2.10	1.97	1.71	1.43	1.43
	IL1RN	3.49	2.06	1.99	1.53	1.47	1.38
	IFNAR2	2.58	1.96	1.24	1.33	1.25	1.31
	IFNGR1	1.38	1.50	1.52	1.49	1.20	1.56
	IL17RA	1.36	1.79	1.44	1.40	1.39	1.29
	IFNGR2	1.02	1.55	1.32	1.38	1.25	1.12
	IL16	0.82	1.74	0.79	1.33	1.01	1.15
	IL4R	1.57	1.70	1.09	0.98	1.11	1.33
enhancer immune genes	GBP1P1	30.48	2.29	26.16	2.06	3.53	1.36
	C4BPA	17.26	2.61	4.61	2.58	2.17	2.03
	ERLIN1	15.81	1.63	2.55	1.65	1.54	1.51
	HERC5	13.62	2.01	5.90	2.03	1.91	1.88
	FCGR1B	10.08	4.48	4.29	2.16	2.48	1.89
	CMPK2	9.53	1.89	6.75	1.97	2.03	1.83
	BATF2	9.35	3.43	21.24	2.19	3.35	1.40
	BMX	9.34	5.70	2.11	1.91	2.07	1.90
	FCGR1A	8.64	3.04	8.20	2.03	2.76	1.41
	IFIT1	8.41	3.36	6.18	2.75	2.23	2.38
	SERPING1	7.69	2.11	12.32	1.99	2.31	1.37
	CD59	7.35	1.69	2.34	1.62	1.44	1.47
	ZC3HAV1	5.88	1.42	2.34	1.59	1.61	1.42
	MX1	5.85	1.77	3.67	1.99	1.71	1.61
	PLEK	5.75	2.40	2.88	1.51	1.69	1.79
	GBP2	5.56	2.52	4.58	1.80	2.16	1.63
	OASL	4.55	2.47	3.84	1.58	1.63	1.71
	MAFF	4.39	1.70	5.06	1.34	1.96	2.12
	ICAM1	3.96	2.95	4.60	1.65	1.72	1.60
	RIPK2	3.86	1.69	2.91	1.35	1.65	1.47
	NMI	2.93	1.72	2.73	1.84	1.68	1.28
	CSNK1A1L	2.91	4.20	1.48	1.92	1.42	2.30
	TRIB1	2.87	2.67	2.26	1.67	1.63	1.68
	NAMPT	2.76	3.61	2.46	1.83	1.80	2.54
	AP5B1	2.23	2.44	2.55	1.49	1.95	1.62
	BCL2A1	1.88	1.60	2.73	1.37	1.81	1.49

Supplementary Table 4. Ratio of reads supporting the reference or mutant sequence at each JAK and STAT mutation site. Black: reference; Red: mutation.

			RNA-seq			WGS		
			%		# of read	%		# of read
Mutations	individual	data	G	T		G	T	
JAK2-V617F	#9	Data-1	21	79	744			deceased
		Data-2	21	79	754			
		Data-3	16	84	675			
		Data-4	15	85	823			
	#10	Data-1	75	25	369	40	60	40
		Data-2	75	25	331			
		Data-3	68	33	440			
Mutations	individual	data	C	T	# of read	C	T	# of read
JAK3-V722I	#2	Data-1	55	45	334	57	43	42
		Data-2	53	47	612			
		Data-3	53	46	515			
		Data-4	56	44	203			
	#26	Data-1	47	53	474	55	45	49
		Data-2	46	54	543			
		Data-3	48	52	340			
		Data-4	52	48	243			
		Data-5	51	49	273			
Mutations	individual	data	G	A	# of read	G	A	# of read
JAK3-L1073F	#13	Data-1	52	48	61	47	53	53
		Data-2	40	60	65			
		Data-3	41	59	106			
		Data-4	47	53	64			
Mutations	individual	data	C	G	# of read	C	G	# of read
STAT2-M594I	#4	Data-1	53	47	996	40	60	42
		Data-2	51	48	3495			
		Data-3	50	50	434			
		Data-4	51	49	1136			
	#15	Data-1	53	47	971	58	42	45
		Data-2	49	51	2041			
		Data-3	50	50	2374			
		Data-4	50	50	1196			
	#16	Data-1	51	49	702	56	41	41
		Data-2	52	48	1193			
		Data-3	47	53	2948			
		Data-4	54	46	602			

			RNA-seq			WGS		
			%		# of read	%		# of read
Mutations	individual	data	C	A		C	A	
STAT2-G825C	#18	Data-1	48	52	1835	51	49	47
		Data-2	51	49	1728			
		Data-3	48	52	2056			
		Data-4	48	52	3611			
		Data-5	49	51	2803			
		Data-6	50	50	1000			
Mutations	individual	data	C	T	# of read	C	T	# of read
TYK2-A53T	#2	Data-1	52	48	295	54	46	28
		Data-2	50	50	510			
		Data-3	48	52	456			
		Data-4	43	56	186			
Mutations	individual	data	C	T	# of read	C	T	# of read
TYK2-G363S	#10	Data-1	44	56	415	35	65	51
		Data-2	48	52	404			
		Data-3	44	56	296			
	#16	Data-1	52	48	415	53	47	45
		Data-2	49	51	749			
		Data-3	45	55	403			
		Data-4	57	43	316			
Mutations	individual	data	A	C	# of read	A	C	# of read
TYK2-I684S	#4	Data-1	53	47	391	39	61	41
		Data-2	58	42	272			
		Data-3	55	45	219			
		Data-4	48	52	277			
	#15	Data-1	46	54	334	46	54	24
		Data-2	51	49	356			
		Data-3	51	49	411			
		Data-4	46	54	287			
Mutations	individual	data	G	C	# of read	G	C	# of read
TYK2-P1104A	#2	Data-1	49	51	1566	51	49	43
		Data-2	50	50	1537			
		Data-3	46	54	1158			
		Data-4	52	48	561			
	#4	Data-1	50	50	1501	42	58	52
		Data-2	48	52	1032			
		Data-3	48	52	922			
		Data-4	53	47	1169			

References

1. Li, H. & Durbin, R. Fast and accurate short read alignment with Burrows-Wheeler transform. *Bioinformatics* **25**, 1754-60 (2009).
2. Broad Institute. Picard. (2016).
3. Bolger, A.M., Lohse, M. & Usadel, B. Trimmomatic: a flexible trimmer for Illumina sequence data. *Bioinformatics* **30**, 2114-20 (2014).
4. Dobin, A. *et al.* STAR: ultrafast universal RNA-seq aligner. *Bioinformatics* **29**, 15-21 (2013).
5. Casper, J. *et al.* The UCSC Genome Browser database: 2018 update. *Nucleic Acids Res* **46**, D762-D769 (2018).
6. Consortium, E.P. An integrated encyclopedia of DNA elements in the human genome. *Nature* **489**, 57-74 (2012).
7. Li, H. Toward better understanding of artifacts in variant calling from high-coverage samples. *Bioinformatics* **30**, 2843-51 (2014).
8. Quinlan, A.R. & Hall, I.M. BEDTools: a flexible suite of utilities for comparing genomic features. *Bioinformatics* **26**, 841-2 (2010).
9. Neph, S. *et al.* BEDOPS: high-performance genomic feature operations. *Bioinformatics* **28**, 1919-20 (2012).
10. Danecek, P. *et al.* The variant call format and VCFtools. *Bioinformatics* **27**, 2156-8 (2011).
11. Anders, S., Pyl, P.T. & Huber, W. HTSeq--a Python framework to work with high-throughput sequencing data. *Bioinformatics* **31**, 166-9 (2015).
12. Love, M.I., Huber, W. & Anders, S. Moderated estimation of fold change and dispersion for RNA-seq data with DESeq2. *Genome Biol* **15**, 550 (2014).
13. Zhao, Y. *et al.* TPM, FPKM, or Normalized Counts? A Comparative Study of Quantification Measures for the Analysis of RNA-seq Data from the NCI Patient-Derived Models Repository. *J Transl Med* **19**, 269 (2021).
14. Risso, D., Ngai, J., Speed, T.P. & Dudoit, S. Normalization of RNA-seq data using factor analysis of control genes or samples. *Nat Biotechnol* **32**, 896-902 (2014).

15. Wickham H. Ggplot2 : Elegant Graphics for Data Analysis. New York: Springer (2009). viii, p.212
16. Vainchenker, W. & Kralovics, R. Genetic basis and molecular pathophysiology of classical myeloproliferative neoplasms. *Blood* **129**, 667-679 (2017).
17. Torres, D.G. *et al.* Molecular landscape of the JAK2 gene in chronic myeloproliferative neoplasm patients from the state of Amazonas, Brazil. *Biomed Rep* **19**, 98 (2023).
18. Riera, L. *et al.* Description of a novel Janus kinase 3 P132A mutation in acute megakaryoblastic leukemia and demonstration of previously reported Janus kinase 3 mutations in normal subjects. *Leuk Lymphoma* **52**, 1742-50 (2011).
19. Yin, C., Sandoval, C. & Baeg, G.H. Identification of mutant alleles of JAK3 in pediatric patients with acute lymphoblastic leukemia. *Leuk Lymphoma* **56**, 1502-6 (2015).
20. Bouchekioua, A. *et al.* JAK3 deregulation by activating mutations confers invasive growth advantage in extranodal nasal-type natural killer cell lymphoma. *Leukemia* **28**, 338-48 (2014).
21. Ehrentraut, S. *et al.* Th17 cytokine differentiation and loss of plasticity after SOCS1 inactivation in a cutaneous T-cell lymphoma. *Oncotarget* **7**, 34201-16 (2016).
22. Elli, E.M. *et al.* Idiopathic erythrocytosis: a germline disease? *Clin Exp Med* **24**, 11 (2024).
23. Zhao, J. *et al.* Benign and Intermediate-grade Melanocytic Tumors With BRAF Mutations and Spitzoid Morphology: A Subset of Melanocytic Neoplasms Distinct From Melanoma. *Am J Surg Pathol* **46**, 476-485 (2022).
24. Dyment, D.A. *et al.* Exome sequencing identifies a novel multiple sclerosis susceptibility variant in the TYK2 gene. *Neurology* **79**, 406-11 (2012).
25. Diogo, D. *et al.* TYK2 protein-coding variants protect against rheumatoid arthritis and autoimmunity, with no evidence of major pleiotropic effects on non-autoimmune complex traits. *PLoS One* **10**, e0122271 (2015).

26. Ma, Y., Shi, N., Li, M., Chen, F. & Niu, H. Applications of Next-generation Sequencing in Systemic Autoimmune Diseases. *Genomics Proteomics Bioinformatics* **13**, 242-9 (2015).
27. Lopez-Isac, E. *et al.* Influence of TYK2 in systemic sclerosis susceptibility: a new locus in the IL-12 pathway. *Ann Rheum Dis* **75**, 1521-6 (2016).
28. Kyogoku, C. *et al.* Lack of association between tyrosine kinase 2 (TYK2) gene polymorphisms and susceptibility to SLE in a Japanese population. *Mod Rheumatol* **19**, 401-6 (2009).
29. Tang, L. *et al.* Genetic association and interaction between the IRF5 and TYK2 genes and systemic lupus erythematosus in the Han Chinese population. *Inflamm Res* **64**, 817-24 (2015).
30. Wang, Y. *et al.* COVID-19 and systemic lupus erythematosus genetics: A balance between autoimmune disease risk and protection against infection. *PLoS Genet* **18**, e1010253 (2022).
31. Lee, Y.H. & Bae, S.C. Association between TYK2 polymorphisms and susceptibility to autoimmune rheumatic diseases: a meta-analysis. *Lupus* **25**, 1307-14 (2016).
32. Dand, N. *et al.* Exome-wide association study reveals novel psoriasis susceptibility locus at TNFSF15 and rare protective alleles in genes contributing to type I IFN signalling. *Hum Mol Genet* **26**, 4301-4313 (2017).
33. Morelli, M. *et al.* HLA-Cw6 and other HLA-C alleles, as well as MICB-DT, DDX58, and TYK2 genetic variants associate with optimal response to anti-IL-17A treatment in patients with psoriasis. *Expert Opin Biol Ther* **21**, 259-270 (2021).
34. Borcherding, D.C., He, K., Amin, N.V. & Hirbe, A.C. TYK2 in Cancer Metastases: Genomic and Proteomic Discovery. *Cancers (Basel)* **13**(2021).
35. El Houdi, M. *et al.* Association study of the JAK/STAT signaling pathway with susceptibility to COVID-19 in moroccan patient and in-silico analysis of rare variants. *Virus Res* **351**, 199509 (2025).
36. Nhung, V.P. *et al.* Host Genetic Risk Factors Associated with COVID-19 Susceptibility and Severity in Vietnamese. *Genes (Basel)* **13**(2022).

37. Zabihi Rizi, F. *et al.* TYK2 single-nucleotide variants associated with the severity of COVID-19 disease. *Arch Virol* **168**, 119 (2023).
38. Canabrava, P.B.E. *et al.* Evaluation of Tyrosine Kinase-2 (TYK2) signaling pathway gene expression and the presence of the single-nucleotide polymorphism rs12720356 in the peripheral blood of patients with severe psoriasis and loss of systemic treatment response. *An Bras Dermatol* **100**, 501165 (2025).
39. Pellenz, F.M. *et al.* Association of TYK2 polymorphisms with autoimmune diseases: A comprehensive and updated systematic review with meta-analysis. *Genet Mol Biol* **44**, e20200425 (2021).
40. Tao, J.H. *et al.* Meta-analysis of TYK2 gene polymorphisms association with susceptibility to autoimmune and inflammatory diseases. *Mol Biol Rep* **38**, 4663-72 (2011).
41. Saevarsdottir, S. *et al.* Multiomics analysis of rheumatoid arthritis yields sequence variants that have large effects on risk of the seropositive subset. *Ann Rheum Dis* **81**, 1085-1095 (2022).
42. Contreras-Cubas, C. *et al.* Catalytically Impaired TYK2 Variants are Protective Against Childhood- and Adult-Onset Systemic Lupus Erythematosus in Mexicans. *Sci Rep* **9**, 12165 (2019).
43. Dai, B. *et al.* TYK2 rs34536443 (P1104A) Variant Suppresses ICAM1-Mediated Inflammation: Insights From Mendelian Randomization and Functional Analyses. *Psoriasis (Auckl)* **15**, 361-372 (2025).
44. Yuan, S. *et al.* Mendelian randomization and clinical trial evidence supports TYK2 inhibition as a therapeutic target for autoimmune diseases. *EBioMedicine* **89**, 104488 (2023).
45. Kerner, G. *et al.* Homozygosity for TYK2 P1104A underlies tuberculosis in about 1% of patients in a cohort of European ancestry. *Proc Natl Acad Sci U S A* **116**, 10430-10434 (2019).
46. Yuan, Y. *et al.* Association Study of Single-Nucleotide Polymorphisms of STAT2/STAT3/IFN-gamma Genes in Cervical Cancer in Southern Chinese Han Women. *Asian Pac J Cancer Prev* **16**, 3117-20 (2015).

47. Kubota, N. & Suyama, M. An integrated analysis of public genomic data unveils a possible functional mechanism of psoriasis risk via a long-range ERRFI1 enhancer. *BMC Med Genomics* **13**, 8 (2020).
48. Berg, V. *et al.* Common origin and somatic mutation patterns of composite lymphomas and leukemias. *Leukemia* **39**, 1960-1971 (2025).
49. Alexeeva, E. *et al.* Safety and efficacy of canakinumab treatment for undifferentiated autoinflammatory diseases: the data of a retrospective cohort two-centered study. *Front Med (Lausanne)* **10**, 1257045 (2023).
50. Brewin, J.N. *et al.* Genetic Analysis of Patients With Sickle Cell Anemia and Stroke Before 4 Years of Age Suggest an Important Role for Apoliprotein E. *Circ Genom Precis Med* **13**, 531-540 (2020).
